# How I perform fertility preservation in breast cancer patients

**DOI:** 10.1016/j.esmoop.2021.100112

**Published:** 2021-04-19

**Authors:** M.G. Razeti, S. Spinaci, F. Spagnolo, C. Massarotti, M. Lambertini

**Affiliations:** 1 Department of Internal Medicine and Medical Specialties (DiMI), School of Medicine, University of Genova, Genoa, Italy; 2 Department of Medical Oncology, UOC Clinica di Oncologia Medica, IRCCS Ospedale Policlinico San Martino, Genoa, Italy; 3 Division of Breast Surgery, Ospedale Villa Scassi, Genoa, Italy; 4 Department of Medical Oncology, Oncologia Medica 2, IRCCS Ospedale Policlinico San Martino, Genoa, Italy; 5 Physiopathology of Human Reproduction Unit, IRCCS Ospedale Policlinico San Martino, Genoa, Italy

## Introduction

Additional age-related issues should be considered when managing breast cancer in young women.^1^ Among them, the potential consequences of anticancer treatments on ovarian function and fertility potential are of particular concern and need to be addressed as early as possible after diagnosis.^2^^,^^3^ Therefore, current guidelines strongly voice the need to provide proper oncofertility counseling with all patients of reproductive age with newly diagnosed cancer irrespective of its stage.^2^^,^^3^ This is a crucial step to discuss with all young women their disease and prognosis, the required treatments including their potential gonadotoxicity, the need for contraception during and after active systemic therapies, their future desire of conception, likelihood of pregnancy, its safety and outcomes as well as the available strategies to avoid the negative impact of the proposed treatment on ovarian function and fertility.^2^^,^^3^

Considering the rising trend in delaying childbearing and the suboptimal knowledge of health care providers towards these survivorship issues,^4^ further awareness and education towards optimizing the oncofertility counseling of young women with breast cancer need to be prioritized.

## Assessing treatment-related gonadotoxicity

To estimate the risk of developing treatment-related premature ovarian insufficiency (POI) and subsequent infertility represents the first important step to achieve during oncofertility counseling.^1^, ^2^ Patient-related and disease/treatment-related factors should be considered to provide a proper estimation of gonadotoxicity risk, with age of the patient at diagnosis and type of therapy being the most important factors.^2^^,^^3^

In young women, there are several issues to be considered when defining POI and infertility. Anticancer therapies can impact female ovarian reserve and reproductive potential beyond possible perturbation in ovarian function.^2^^,^^3^ Several studies are evaluating the clinical utility to assess serum concentrations of anti-Mullerian hormone (AMH) before, during and after treatment, to better estimate the risk of gonadotoxicity.^2^^,^^3^ A better indicator of treatment effect on women’s ovarian reserve would be crucial for improving this first step of oncofertility counseling. In addition, a better indicator of the gonadotoxic impact of chemotherapy would be of particular relevance in young patients with hormone receptor-positive breast cancer, for whom a proper assessment of their ovarian function after the completion of cytotoxic therapy is needed also to optimize adjuvant endocrine therapy.^5^

Several studies have reported the gonadotoxicity of the most commonly used chemotherapy regimens in premenopausal women with breast cancer using both amenorrhea rates and AMH levels following its administration as the definition of treatment-related POI (Table 1).^2^

**Table 1:** Estimated risk of treatment-related amenorrhea in breast cancer patients receiving systemic anticancer therapies

Degree of risk	Treatment type/regimen	Comments
High risk (>80%)	• 6 Cycles of CMF, CEF, CAF or TAC in women aged ≥40 years	Significant decline in AMH concentrations after treatment
Intermediate risk (20%-80%)	• 6 Cycles of CMF, CEF, CAF or TAC in women aged 30-39 years • 4 Cycles of AC in women aged ≥40 years • 4 Cycles of AC/EC → taxane • 4 Cycles of dd (F)EC → dd taxane	Significant decline in AMH concentrations after treatment
Low risk (<20%)	• 6 Cycles of CMF, CEF, CAF or TAC in women aged <30 years • 4 Cycles of AC in women aged <40 years	Significant decline in AMH concentrations after treatment
Very low or no risk	• Antimetabolites (methotrexate, fluorouracil)	
	• Vinca alkaloids	
	• Tamoxifen	No change in AMH concentrations after treatment
	• Bevacizumab (?)	
	• Trastuzumab, lapatinib and T-DM1 (?)	
Unknown/unclear risk	• Platinum- and taxane-based chemotherapy • Most targeted therapies (including monoclonal antibodies and small molecules): pertuzumab, everolimus, CDK4/6 inhibitors, PARP inhibitors • Immunotherapy • GnRHa plus an aromatase inhibitor	

Whenever available, data on post-treatment AMH concentrations, that most accurately reflect the potential damage on the ovarian reserve, are reported as commentary.

AC, doxorubicin, cyclophosphamide; AMH, anti-Mullerian hormone; CAF, cyclophosphamide, doxorubicin, 5-fluorouracil; CDK4/6, cyclin-dependent kinase 4/6; CEF, cyclophosphamide, epirubicin, 5-fluorouracil; CMF, cyclophosphamide, methotrexate, 5-fluorouracil; dd, dose dense; EC, epirubicin, cyclophosphamide; GnRHa, gonadotropin-releasing hormone agonist; PARP, poly (ADP-ribose) polymerase; TAC, docetaxel, doxorubicin, cyclophosphamide.

The highest gonadotoxic risk in breast cancer is associated with the use of the alkylating agent cyclophosphamide and its dose.^2^^,^^3^

In premenopausal women with hormone receptor-positive breast cancer, endocrine therapy use for 5-10 years can indirectly affect the ovarian reserve and fertility potential through aging.^5^ In addition, use of tamoxifen following chemotherapy completion can increase the risk of treatment-related amenorrhea, but does not appear to impact the ovarian reserve.^2^^,^^3^

Limited evidence exists on the gonadotoxicity of targeted therapies and no data exist on immune checkpoint inhibitors. A potential negative effect of bevacizumab cannot be excluded, while the anti-human epidermal growth factor receptor 2 agents trastuzumab, lapatinib and T-DM1 appear to be safe for the ovaries.^2^^,^^3^ However, no strong conclusions can be made in this regard.

Collecting prospective information about the functional and endocrinological impact of modern anticancer therapies on women’s gonadal reserve and reproductive outcomes should become a crucial and vital component of drug development.^6^

Age at diagnosis is the most important patient-related factor to be considered when estimating the risk of gonadotoxicity with the proposed treatment.^2^^,^^3^ Pretreatment ovarian reserve measured by AMH concentration (which is strongly related to age) influences the risk of developing treatment-induced POI. Hereditary conditions, and specifically carrying germline pathogenic variants in the *BRCA* genes, may negatively impact ovarian reserve and performance of fertility preservation strategies, but data are too limited and controversial to conclude about a potential increased risk of treatment-related POI in these patients.^7^ The effect of other factors (including body mass index, smoking history and genetic variants in terms of single-nucleotide polymorphisms) remains to be fully elucidated.^2^^,^^3^

## Strategies for preservation of fertility and/or ovarian function

### Oocyte/embryo cryopreservation

Cryopreservation of oocytes and/or embryos is a standard strategy for fertility preservation and the first option to discuss with all women whenever the ovarian reserve is adequate, a vaginal ultrasound is possible and at least 2 weeks are available before starting anticancer therapy.^2^^,^^3^ This interval is needed for carrying out ovarian stimulation with gonadotropins before vaginal follicle aspiration followed by cryopreservation (for oocytes) or fertilization and then cryopreservation (for embryos). With ‘random start stimulation’ protocols, ovarian stimulation can also be initiated any time during the menstrual cycle.^2^^,^^3^

The success of this strategy is strongly dependent on the number of mature oocytes collected after ovarian stimulation, which is directly linked with the age of the patient and her ovarian reserve at diagnosis.^2^^,^^3^ A live birth rate >40% can be estimated in women younger than 35 years, and <30% in older patients, with a very low success after the age of 40 years.^8^ Although there is no apparent negative influence of breast cancer diagnosis on the success of the procedure, some evidence suggests a potential reduced performance of oocyte/embryo cryopreservation in breast cancer patients carrying germline *BRCA* pathogenic variants.^7^ However, oocyte/embryo cryopreservation remains the first option to be discussed also in *BRCA*-mutated breast cancer patients.^2^ Importantly, this strategy allows access to preimplantation genetic testing that can be of importance for these women.^7^

Safety concerns have been raised with the use of ovarian stimulation for approximately 2 weeks in women newly diagnosed with breast cancer and particularly in those with hormone receptor-positive disease.^4^ However, despite the limited evidence, available data support the safety of carrying out ovarian stimulation after diagnosis and before starting adjuvant or neoadjuvant chemotherapy.^2^^,^^3^ The addition of letrozole during ovarian stimulation may help to reduce estradiol concentrations without any apparent negative effect on the efficacy of the strategy.^9^ Therefore, the inclusion of letrozole in the protocols for ovarian stimulation in breast cancer patients may be considered the preferred approach.^2^^,^^3^

### Ovarian tissue cryopreservation

Ovarian tissue cryopreservation is an alternative approach to preserve fertility (and ovarian function) when oocyte/embryo cryopreservation is not feasible.^2^^,^^3^ It consists of ovarian cortex biopsies or unilateral ovariectomy usually carried out by laparoscopy under general anesthesia, followed by cryopreservation. Thawing and subsequent transplantation (most often done orthotopically to allow spontaneous pregnancies) can be carried out any time following anticancer treatment completion if there is no spontaneous resumption of ovarian function or natural conception. Importantly, this strategy does not require ovarian stimulation and anticancer treatments can be started the day after the procedure.^2^^,^^3^

There are two crucial factors for the success of ovarian tissue cryopreservation: the expertise of the laboratory in cryopreserving the tissue and the age of the patient at the time of the procedure. With a ‘hub and spoke’ model, regional networks should be implemented to optimize the success of this strategy, with the possibility to carry out the surgery locally while referring the tissue to a central experienced facility for cryostorage.^2^^,^^3^ In terms of age, only a few pregnancies have been obtained in women older than 36 years, thus supporting this age as the cut-off point to propose ovarian tissue cryopreservation.^2^^,^^3^ With these caveats, a live birth rate of around 40% is expected, with approximately half of the pregnancies being spontaneous.^10^

In terms of safety, the risks and complications related to the procedure are low in women without contraindication to surgery/anaesthesia.^2^^,^^3^ In women with early breast cancer, the risk of disease transmission during transplantation due to residual neoplastic cells within the ovarian cortex is considered low, but it is crucial to use adequate techniques to exclude malignant contamination of the cryopreserved tissue.^2^^,^^3^ Caution is needed in women at increased risk of ovarian cancer due to hereditary predisposition (e.g. because of germline *BRCA* pathogenic variants). Limited evidence is available in this setting.^7^ However, ovarian tissue cryopreservation carried out years before the recommended age of risk-reducing gynecological surgery is not contraindicated *per se*, although specific considerations are needed including the choice of the transplantation site.^2^^,^^3^

### Ovarian suppression with a gonadotropin-releasing hormone agonist during chemotherapy

In premenopausal breast cancer patients, medical gonadoprotection obtained by administering gonadotropin-releasing hormone agonist (GnRHa) during chemotherapy is standard strategy for ovarian function preservation.^2^^,^^3^ This option aims at reducing POI risk and all its associated endocrine-related side-effects. Therefore, it can be considered highly relevant also to women without pregnancy desire and not interested in fertility preservation strategies. GnRHa use during chemotherapy should not be considered as a stand-alone fertility preservation strategy but can be used in conjunction with cryopreservation options.^2^^,^^3^

A large meta-analysis based on individual patient-level data from 873 patients randomized in five major breast cancer trials reported the efficacy and safety of this strategy.^11^ Administering GnRHa during chemotherapy significantly reduces POI rates [from 30.9% to 14.1%; adjusted odds ratio (OR) 0.38; 95% confidence intervals (CI) 0.26-0.57]. Treatment benefit was observed in both patients younger or older than 40 years and in both women with hormone receptor-positive and -negative breast cancer. Despite the low numbers, more post-treatment pregnancies were observed in women treated with GnRHa during chemotherapy compared with those who received cytotoxic therapy alone (37 versus 20 pregnancies; incidence rate ratio 1.83, 95% CI 1.06-3.15).^11^

Concurrent use of GnRHa during chemotherapy was shown to be safe with similar disease-free survival [adjusted hazard ratio (HR) 1.01; 95% CI 0.72-1.42] and overall survival (adjusted HR 0.67; 95% CI 0.42-1.06) in patients treated with or without pharmacological ovarian suppression during cytotoxic therapy. The safety of this strategy was observed irrespective of hormone receptor status.^11^ However, in premenopausal women with hormone receptor-positive breast cancer, subsequent ovarian function suppression should be considered as adjuvant endocrine therapy for most of these patients previously exposed to chemotherapy.^1^^,^^5^

## Pregnancy after breast cancer

Breast cancer survivors have a significantly reduced likelihood of having a subsequent pregnancy compared with the general population, with an estimated 60% lower prevalence (relative risk 0.40; 95% CI 0.32-0.49).^12^ Possible explanations for these findings are the concerns shared by both patients and their health care providers that a prior exposure to systemic anticancer treatment may negatively affect the pregnancy itself (by increasing the risk of complications) and that conceiving in women with a prior history of breast cancer may potentially have a detrimental prognostic effect (by increasing the risk of recurrence), particularly in the case of hormone receptor-positive disease.^4^

These concerns have been recently dispelled by a large systematic review and meta-analysis.^12^ No alarming signals were observed for the majority of the analyzed reproductive outcomes including no significant increased risk of congenital abnormalities. However, compared with the general population, breast cancer patients showed an increased risk of undergoing caesarean section (OR 1.14; 95% CI 1.04-1.25), to have offspring with low birth weight (OR 1.50; 95% CI 1.31-1.73), preterm birth (OR 1.45; 95% CI 1.11-1.88) and small for gestational age (OR 1.16; 95% CI 1.01-1.33). The risk of developing these complications, which appeared to be mostly restricted to patients previously exposed to chemotherapy, suggests the need for a close monitoring of these pregnancies in experienced units.^12^

In terms of patients’ prognosis, no detrimental effect of pregnancy after breast cancer was shown, even when correcting for the potential guarantee-time bias (including the possible ‘healthy mother effect’). Patients with a post-treatment pregnancy showed a better disease-free survival (HR 0.73; 95% CI 0.56-0.94; HR adjusted for guarantee-time bias 0.74; 95% CI 0.58-0.96) and overall survival (HR 0.56; 95% CI 0.46-0.67; HR adjusted for guarantee-time bias 0.52; 95% CI 0.42-0.65) compared with women with breast cancer without subsequent pregnancy. The safety of pregnancy was observed irrespective of patient, tumor, treatment characteristics, timing and outcome of pregnancy.^12^

Based on the reassuring growing body of knowledge on the topic, pregnancy in women with a prior history of breast cancer should not be discouraged following proper treatment and follow-up.^2^^,^^3^ The prospective POSITIVE trial (NCT02308085) has recently completed accrual and will report on the safety of a temporary interruption of adjuvant endocrine therapy to attempt pregnancy in women with hormone receptor-positive breast cancer.^13^

## Conclusions

Nowadays, giving hope for a family after cancer diagnosis and treatment should be considered a crucial ambition in cancer care.^14^ Therefore, oncofertility care has become a priority and a mandatory component of the management of young women with newly diagnosed breast cancer.^2^^,^^3^ Increased awareness by all health care professionals in oncology is needed to make sure this topic is always discussed at diagnosis and women can make fully informed decisions about the proposed anticancer therapies and their potential interest in accessing the available strategies for ovarian function and/or fertility preservation.^2^^,^^3^

**Figure 1:**
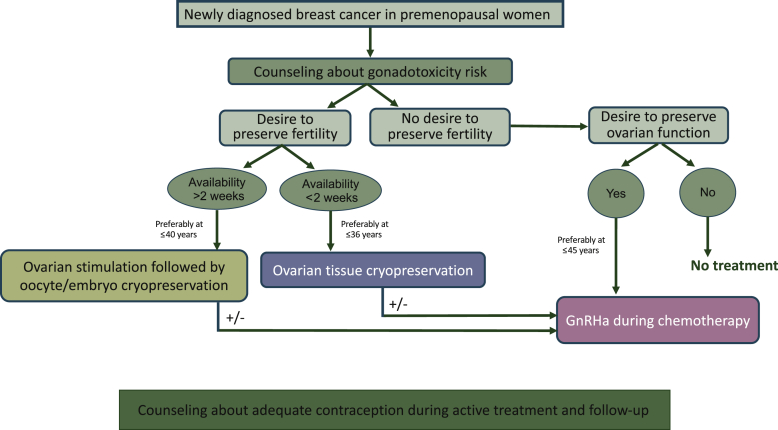
Oncofertility counseling in premenopausal women with breast cancer. GnRHa, gonadotropin-releasing hormone agonist.

Building a strong oncofertility network within or outside the cancer center is the first crucial step to accomplish for allowing a proper path to all patients in this setting (Figure 1). Notably, this collaboration between oncologists and fertility specialists is not only important at diagnosis for women interested in ovarian function and/or fertility preservation. In fact, it has been shown to be also crucial during oncological follow-up in order to properly manage the gynecological side-effects of anticancer therapies, as well as to discuss other issues (e.g. contraception) of great importance for all patients and particularly for women with hormone receptor-positive disease receiving adjuvant endocrine therapy.^15^

Despite many studies have been carried out over the past years in this field, additional prospective research efforts are needed to further improve and ensure optimal oncofertility care in young women with early breast cancer.
